# Towards reliable elastic characterization of glass bead reinforced thermoplastic composites using impulse excitation and conventional testing

**DOI:** 10.1038/s41598-026-36346-z

**Published:** 2026-01-22

**Authors:** Julian Rech, Christian Dresbach, Esther Ramakers van Dorp, Bernhard Möginger, Berenika Hausnerova

**Affiliations:** 1 Bonn-Rhein-Sieg University of Applied Sciences, von-Liebig-Straße 20, 53359 Rheinbach, Germany; 2 Centre of Polymer Systems, University Institute, Tomas Bata University in Zlin, nam. T.G. Masaryka 5555, Zlin, 76001 Czech Republic; 3 Faculty of Technology, Tomas Bata University in Zlin, Vavreckova 275, Zlin, 76001 Czech Republic

**Keywords:** Composite, Elastic constant, Impulse excitation technique, Tensile testing, Dynamic mechanical analysis, Oscillatory torsion, Engineering, Materials science

## Abstract

Reliable determination of elastic properties is essential for the structural use of polymer composites in engineering applications. This work aims to evaluate the impulse excitation technique (IET) as a method for determining elastic constants of glass bead‑reinforced polyamide 66 (PA66) and polybutylene terephthalate (PBT), and to compare its performance to tensile testing (TT), dynamic mechanical analysis (DMA), and oscillatory torsion (OT). Commercial PA66 and PBT grades with 0–40 wt% glass beads were injection‑molded and annealed; the addition of glass beads increased Young’s moduli by 60–70% for PA66 and 40–60% for PBT compared to the neat matrices, depending on filler content. IET, supported by finite element analysis, provided dynamic flexural and longitudinal moduli, shear modulus, and Poisson’s ratio which were comparable to those obtained from TT, DMA and OT. In the linear elastic regime IET data differed within the standard deviations of TT, DMA and OT. The moduli determined by flexural excitation using IET and DMA agreed within experimental uncertainty only above an amplitude threshold of approximately 110 μm, while Young’s moduli from TT and IET showed good agreement, though TT exhibited greater variability. Consistent trends were also found for shear modulus and Poisson’s ratio. The higher longitudinal moduli (4 to 8% for PA66 and 2 to 4% for PBT) measured by IET are explained by higher frequencies (3 to 4 orders of magnitude) and cross-sectional microstructural anisotropy and crystallinity differences confirmed by microscopy and calorimetry. The results demonstrate that IET is a much faster, non‑destructive and accurate method for obtaining elastic constants of thermoplastic composites particularly suited for the design and dimensioning of load‑bearing structural components.

**Supplementary Information:**

The online version contains supplementary material available at 10.1038/s41598-026-36346-z.

## Introduction

With the growing demand for durable and lightweight materials in structural engineering, the mechanical properties of polymer matrix composites (PMC) have become critical for the design and dimensioning of load-bearing components. While PMCs are well established in sectors such as automotive, aerospace, and maritime industries, their use is expanding into civil engineering and construction (e.g. modular building elements and infrastructure components), where mechanical performance and durability are essential. In parallel, sustainability considerations are becoming increasingly important, prompting the use of recycled polymers for PMC compounding. These recycled-based systems, however, often differ significantly in mechanical and physical behavior compared to virgin compounds^1^.

For structural applications, accurate knowledge of stiffness-related parameters such as the static Young’s modulus, dynamic storage modulus, complex modulus, and shear modulus is vital. These elastic constants directly influence load-bearing capacity, deformation limits, and long-term performance of PMC. Their determination can be achieved either through experimental testing using various mechanical techniques, or by theoretical prediction via finite element analysis (FEA), which enables estimation of effective bulk properties based on constituent phase behavior and material architecture. In addition to the elastic properties of PMC, literature clearly highlights their advantageous behavior. Numerous studies show how filler shape and size alter material behavior e.g. tensile properties of spherical shape silicon carbide reinforced recycled polyethylene^2^, thermal, mechanical, and shape memory behavior of thermo-responsive polyurethane nanocomposites^3^, or bulk mechanical properties of 1D/2D nanofiller reinforced polyethylene^4^.

Various measurement techniques have been used for the experimental determination of properties. Many scientists have studied the effect of matrix type and dispersed phase on the Young´s modulus for different particle sizes, filler volume contents *v*_F_, and filler/matrix adhesion using tensile testing (TT). Polymer matrices behave isotropically, which is true for randomly oriented polymer chains^5^. However, during processing, time- and location-dependent shear rates, varying cooling rates, local filler accumulation, or different particle volume contents occur, leading to flow-direction-dependent chain orientations or skin-core structures. These morphologies exhibited different mechanical properties over the cross-sections. Serra-Aguila et al.^6^ determined the longitudinal (l) Young´s modulus *E*_l_ of polyamide 66 (PA66) using TT at a displacement rate of 1 mm/min and dynamic flexural (*f*) storage modulus *E*´_f_ using dynamic mechanical analysis (DMA) in 3-point bending at varying frequencies corresponding to displacement rates ranging from 1 to 20 mm/min. They found that static *E*_l_ exceeded dynamic *E*´_f_ by a factor of 1.2. Assuming isotropic material properties, an analytical approach was applied to convert the moduli into each other for varying strain rates and temperatures; however, the differences between these values were not discussed. Unal et al.^7^ compared the mechanical properties of longitudinal and flexural static loading of polyamide 6 (PA6) filled with various amounts of spherical glass beads, flake-like kaolin, talc, and fibrous wollastonite. Both $\:{E}_{\mathrm{l}}$ and $\:{E}_{\mathrm{f}}$ increased with increasing filler content and aspect ratio with approx. two times higher $\:{E}_{\mathrm{l}}$ over the entire filler volume range for all fillers, indicating inhomogeneous mechanical behavior due to skin-core arrangements. An opposite trend was observed by Savas et al.^8^ for PA6 filled with ceramic microspheres. While the longitudinal moduli varied only between 1.1 and 1.4 GPa at weight contents ranging from 10 to 40 wt%, respectively, the flexural modulus varied between 2.5 and 4.0 GPa. For a low increase in $\:{E}_{\mathrm{l}}$ they concluded that the increase in filler particles is compensated by a decrease in crystallinity and a higher crack propagation probability. They also implied that for $\:{E}_{\mathrm{f}}$, the high rigidity of ceramic particles compensated for the reduction in the crystallinity for flexural excitation owing to the compression, shear, and tensile forces, where the crack propagation is not a key factor for longitudinal excitation. Guglani et al.^9^ reported similar findings for a PA66 filled with 2–8 wt% titanium dioxide particles, where the flexural moduli were 2.5 times higher than the tensile moduli without any explanation why this inhomogeneity occurs. Furthermore, Deng et al.^10^ and Selvaraj et al.^11^ also found higher moduli for unidirectional deformation than for bending deformation of epoxy and vinyl ester polymer composites. Junaedi et al.^12^ reported that the mechanical behavior of reinforced polypropylene (PP) depends on the loading direction. Injection-molded test bars of neat PP and composites containing particles with different aspect ratios (milled short carbon fibers, graphite nanoplatelets, and titanium dioxide nanoparticles) were investigated using 3-point bending and tensile tests. Both the raw material and composites showed lower $\:{E}_{\mathrm{f}}$ than $\:{E}_{\mathrm{l}}$ independent of the aspect ratio. This indicates inhomogeneous mechanical behavior due to shear flow-induced orientations of the fillers and chain orientations yielding skin-core arrangements. Opposite effects were observed for platelet talc and spherical calcium carbonate-filled acrylonitrile butadiene styrene composites^13^. The injection molding-induced orientations and accumulation of the particles in the skin region caused higher $\:{E}_{\mathrm{f}}$ compared to $\:{E}_{\mathrm{l}}$. This behavior was more pronounced for the composites containing talc owing to its higher aspect ratio, and thus, the higher contribution of the stress transfer in the skin region. Another study by Rothenhäusler et al.^14^ investigated the mechanical behavior of epoxy resins cured with various agents, resulting in different network structures. Although the mechanical properties were considerably influenced by the resulting cross-link density covering a wide stiffness spectrum (2.6–3.7 GPa), all epoxy variants exhibited longitudinal moduli approximately 200 MPa lower than those of the flexural variants. Huayamares et al.^15^ compared the viscoelastic properties of quasi-isotropic glass and carbon fiber-reinforced and unidirectional glass fiber-reinforced epoxies using DMA in 3-point bending and a rheometer in oscillatory torsion (OT). They found a continuously higher $\:{E}_{\mathrm{f}}^{\prime \mathrm{D}\mathrm{M}\mathrm{A}}$ compared with the moduli calculated from the shear modulus *G* in oscillatory torsion tests. Their conclusion was a lack of proportionality through the Poisson´s ratio between *G* and *E´* for the investigated materials. Fauziyah et al.^16^ demonstrated that a filler morphology also plays an important role; thermomechanical dynamic tensile and shear moduli of composites containing 20 wt% of different silica polymorphs (quartz, cristobalite, and amorphous) in poly(ethylene glycol) (PEG) increased 12 times for PEG/quartz, 10 times for PEG/cristobalite, and 11 times for PEG/amorphous phase compared to neat PEG. Further studies investigated the capability of natural fiber reinforced epoxy systems^17,18^.

From the literature, it is clear that the elastic parameters obtained from tensile testing, dynamic mechanical analysis, and oscillatory shear tests vary nonuniformly in the excitation direction. In this work, an impulse excitation technique (IET), which is well-established for ceramics and metals^19–22^ but is omitted in the area of thermoplastic composites, is considered. It enables the measurement of longitudinal, flexural, and shear moduli, as well as the Poisson ratio, in a non-destructive manner. IET was previously used for stiff PMC, especially dental materials with filler volume contents of up to 90 vol%^23–25^. Tognana et al.^26^ investigated epoxy composites containing 5 to 30 vol% of aluminum, quartz, and copper particles. A flexural setup was selected to obtain the resonant frequencies that provided the flexural moduli. Subsequently, the IET moduli were compared with the calculated moduli according to the analytical approaches of Hashin-Shtrikman^27^ and Kerner^28^. In particular, the moduli of the copper-containing composites were in good agreement with the calculated moduli, owing to their spherical shape. Pittala et al.^29^ compared the flexural moduli obtained from standard flexural tests for neat epoxy resin and epoxy resin filled with carbon fibers. They determined 1.2 (for neat epoxy) and 1.1 (for composite) times higher IET moduli than those determined with 3-point bending attributing these differences to strain rate effects and intrinsic inhomogeneities. Furthermore, they noted that the elastic modulus obtained through IET represents the overall elastic modulus, whereas the modulus determined through flexural tests depends mainly on local strain effects, and represents the local elastic modulus.

Notably, much effort was spent to prove that IET is an excellent method to determine elastic properties for a wide variety of materials^30,31^. All aforementioned studies show that the elastic behavior of polymers and composites is time-dependent and influenced by complex interactions during the manufacturing process, filler aspect ratio, filler volume content, and polymer type. However, systematic quantitative studies using IET for thermoplastic composites as well as their validation with respect to standardized other mechanical tests are largely lacking. Towards reliable property generation, this study aims to provide a comprehensive and experimentally validated application of IET for thermoplastic glass bead composites to determine their elastic properties for defined conditions. The findings from non-destructive IET are quantitatively compared to more time-consuming and demanding methods such as tensile testing, DMA, and oscillatory torsion, accompanied by a full error analysis and assessment of accuracy and limitations.

## Materials and methods

### Materials and their characterization

Injection molded test bars (type 1 A according to ISO 527 2) of two commercially available polymers, polyamide 66 (PA66) and polybutylene terephthalate (PBT), filled with glass beads (GB) (Table 1) were used to determine elastic properties. In this study the GB content was varied to investigate composites with different stiffnesses to assess deviations of different testing methods. The size of GB and GB/matrix bonding were kept unmodified.

**Table 1:** List of PMC showing matrix polymer, brand name, GB content/diameter, young’s modulus, glass temperature and density according to data sheets.

Matrix	Brand name	GB content	GB diameter	Young’s modulus	Glass temperature	Density
		*w*_F_ [%]	*D* [µm]	*E* [MPa]	*T*_g_^4^ [°C]	*ρ* [g/cm³]
PA 66	RADIPOL^1^ A45	0	-	3200	58	1.14
	AKROMID^2^ A3 GK 30	30	25–40	5000	55	1.35
	AKROMID A3 GK 40	40	25–40	5500	63	1.44
PBT	Ultradur^3^ B 2550	0	-	2500	43	1.30
	Ultradur B 4300 K4	20	25–40	3500	43	1.45
	Ultradur B 4300 K6	30	25–40	4000	42	1.53

^1^ brand name of Radici Group, Gandino, Italy^32^ ^2^ brand name of AKRO-PLASTIC GmbH, Niederzissen, Germany^33,34^ ^3^ brand name of BASF SE, Ludwigshafen, Germany^35–37^ ^4^ $\:{T}_{\mathrm{g}}$ was determined by DMA at a frequency of 1 Hz as the mean of $\:{T}_{\mathrm{g}}^{\mathrm{o}\mathrm{n}\mathrm{s}\mathrm{e}\mathrm{t}}$ and $\:{T}_{\mathrm{g}}^{\mathrm{e}\mathrm{n}\mathrm{d}\mathrm{s}\mathrm{e}\mathrm{t}}$.

**Fig. 1:**
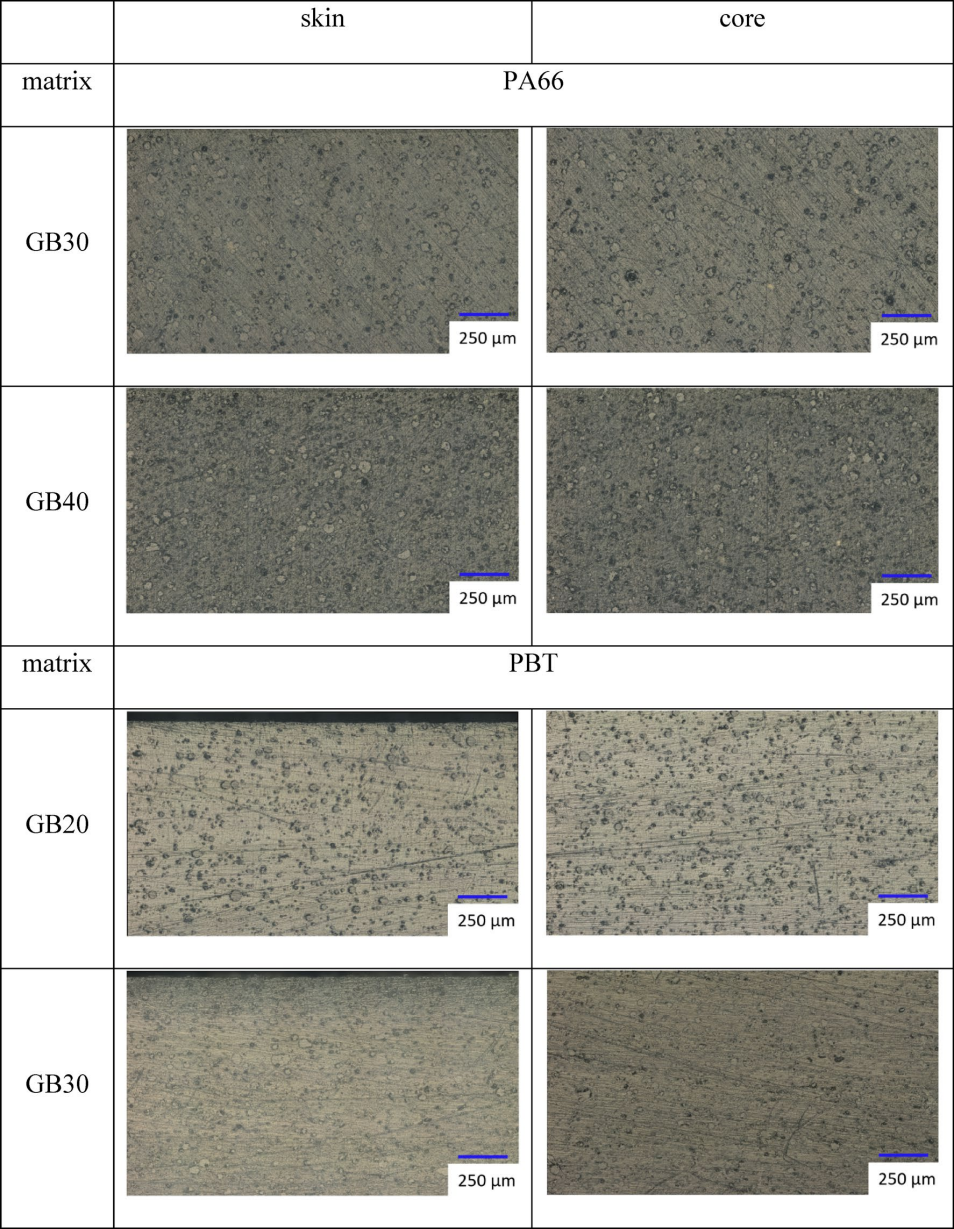
GB distribution in PA66 and PBT composites cross-sections.

**Fig. 2:**
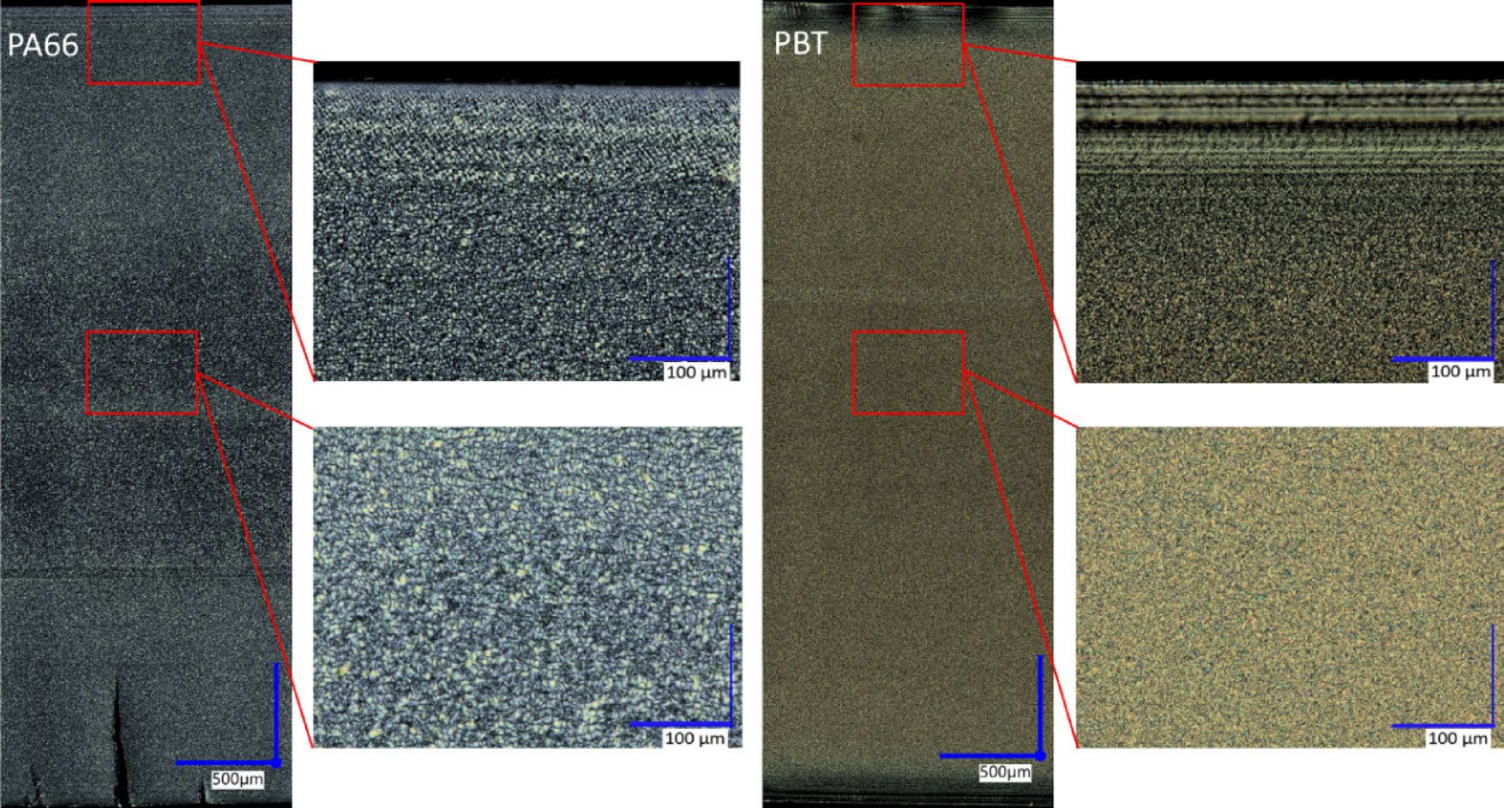
Skin core morphology of annealed PA66 (left) and PBT (right) along the cross-section; flow direction ↔.

The cross-sectional distribution of GB was investigated for both PMC to check for processing-induced inhomogeneities, Fig. 1. A digital microscope (VHX7000, Keyence, Osaka, Japan) was used to map the polished cross sections of the PA66 and PBT composites under reflected light microscopy. Polarized light and the high dynamic range function were applied at magnifications of 200x and 500x to check for cross-sectional GB distributions. This was done by counting the GB in steps with a step-width area of (200 × 2000) µm. Furthermore, the morphology of the neat material induced by injection molding was investigated under transmitted light microscopy with the same microscope, Fig. 2. Thin sections (thickness *t* = 15 μm) were cut from the specimens after annealing using a microtome (Leica RM2165, Leica Biosystems, Nussloch, Germany). Thereafter, the thin sections were embedded in Canada balsam under a cover glass on a microscope slide and investigated under polarized light. The whole cross-sections were investigated at magnifications of 500x and 700x to examine the morphology of neat PA66 and PBT.

Both PMC exhibited only slightly pronounced decay in GB concentrations in the skin regions, whereas they were homogeneous in the core (Fig. 1 and **Appendix A1)**. Concentration differences of GB between skin and core were determined as 5% for PA66 GB30, 7% for PA66 GB40, 3% for PBT GB20, and 6% for PBT GB30. Assuming identical matrix moduli in both the skin and core, the reduced GB content decreases the moduli of the PMC by approximately 300 MPa for PA66 GB30, 400 MPa for PA66 GB40, 200 MPa for PBT GB20, and 300 MPa for PBT GB30 at a constant filler matrix adhesion^38^. It should be noted that the microscopic analysis in this work was limited to optical microscopy of polished cross sections providing qualitative and semi-quantitative insight into glass bead distribution. This approach was sufficient to identify processing-induced inhomogeneities relevant for interpreting differences between flexural and longitudinal elastic moduli. However, it does not resolve interfacial features, local strain fields, or statistically detailed filler distributions as more comprehensive microstructural characterization, which represents an important direction for future work.

The morphology induced by injection molding is shown in Fig. 2. The thickness of the skin layer was of the order of 200 μm for both polymer matrices, indicating that the cooling conditions were similar. The smaller spherulites of PBT can be attributed to their higher nucleation and crystallization rates^39,40^. Because the skin layers solidify faster owing to the rapid temperature decrease, the crystallinity is lower than that of the core. Thus, one must expect a cross-section-dependent distribution of internal stresses and more crystallization-dependent shrinkage in the core. As the macroscopic strain state of the test bars is fixed, compression stresses in the skin layers and tensile stresses in the core region are expected. Another manufacturing effect is that Young’s moduli decrease in the flow direction of the melt along the length of the test bars^41^. Therefore, all test bars were annealed at 180 °C prior to mechanical and thermal testing to establish a uniform thermal history.

DSC measurements were performed using a differential scanning calorimeter (DSC 214 Polyma; Netzsch, Selb, Germany). Thin samples of approximately 10 mg were cut from both the skin and core regions parallel to the length direction of the dumbbell specimen. The samples were placed in aluminum crucibles and sealed with pierced lids. The PMC of PA66 was measured in the temperature range 30–300 °C, and the PMC of PBT in the temperature range 25–260 °C at a heating rate of 10 K/min under nitrogen atmosphere (20 ml/min). The crystallinity *X*_C_ was evaluated using Eq. (1):

1 $$\:{X}_{C}=\frac{\varDelta\:{h}_{\mathrm{m}}-{\varDelta\:h}_{\mathrm{p}\mathrm{c}}}{(1-{w}_{\mathrm{F}})\varDelta\:{h}_{\mathrm{m}}^{0}}$$

with measured heat of fusion $\:\varDelta\:{h}_{\mathrm{m}}$, heat of fusion of a 100% crystalline polymer $\:\varDelta\:{h}_{\mathrm{m}}^{0}$ taken from^42,43^, post-crystallization enthalpy $\:{\varDelta\:h}_{\mathrm{p}\mathrm{c}}$, and filler weight content *w*_F_.

Melting temperatures $\:{T}_{\mathrm{m}}$ and post-crystallization temperatures $\:{T}_{\mathrm{p}\mathrm{c}}$ were evaluated with respect to the zero point of the first derivative. The enthalpies were evaluated between 180 and 290 °C and 180–250 °C for PA66 and PBT composites, respectively, using a linear baseline function. Test bars were annealed for 4 h at 180 °C, just below the crystallization temperatures of PBT (195 °C) and PA66 (230 °C) and significantly above the glass transition temperatures of PBT (50 °C) and PA66 (75 °C). This annealing process not only deletes a large part of the thermal history due to processing, but also increases the crystallinity of the test bars due to post-crystallization.

**Fig. 3:**
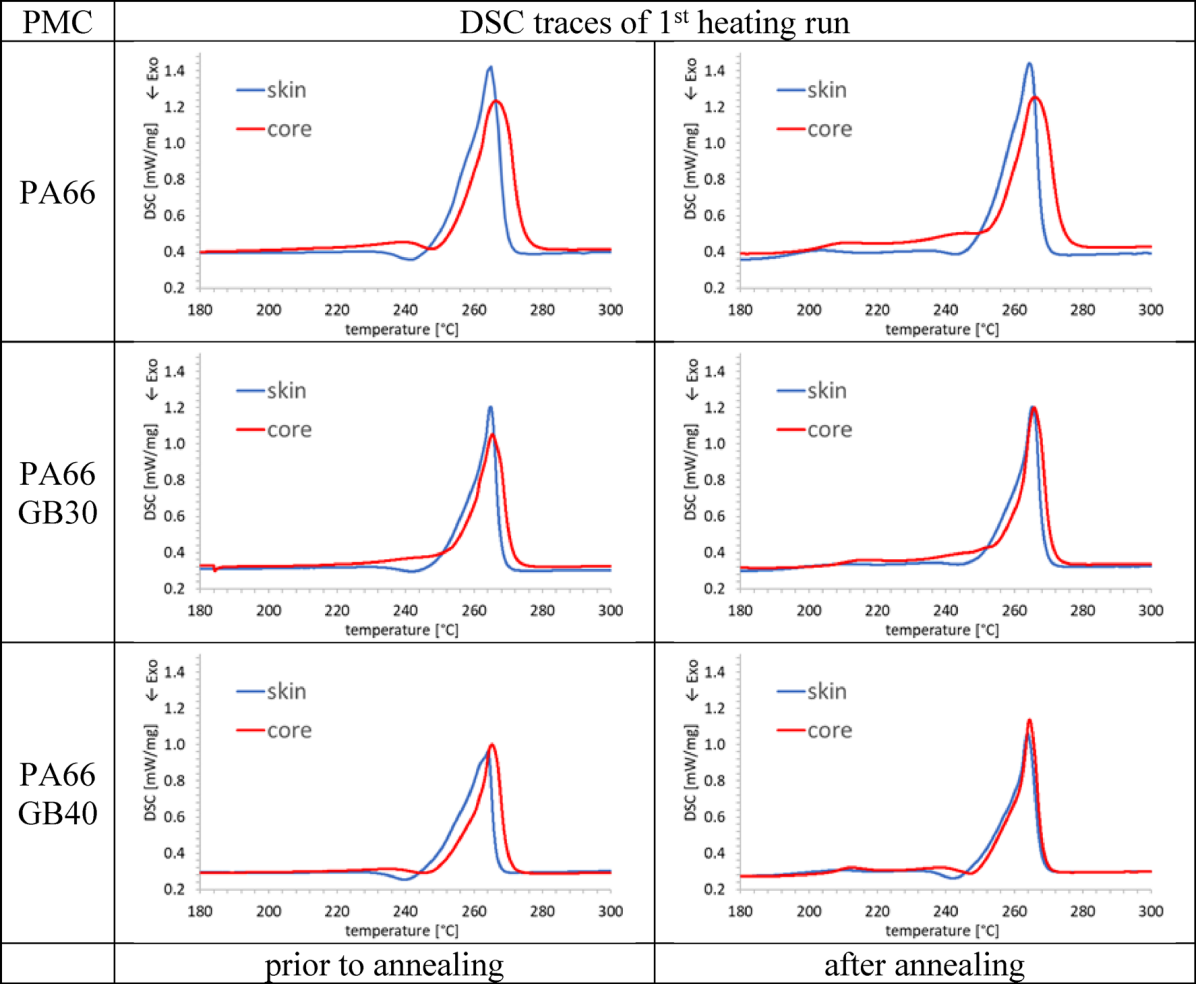
Effects of annealing for 4 h at 180 °C on DSC traces of PA66 PMC, and reduction of heats of fusion with increasing GB content.

The DSC traces of all PA66 PMC exhibited differences in the initial state between skin and core, Fig. 3. The post-crystallization peaks are clearly visible for the skin region around 240 °C and are less pronounced or even not occurring for the core region. The melting peaks were observed at lower temperatures compared to the core regions, indicating that the thicknesses of the crystalline lamellae of the core regions exceeded those of the skin regions; in particular, the heats of fusion were larger for the core regions, Table 2.

**Table 2:** Crystallization and melting behavior of PA66 composites and PBT composites before and after annealing.

Parameter			Polymer Matrix Composite					
			PA66			PBT		
			*w*_F_ [%]			*w*_F_ [%]		
			0	30	40	0	20	30
*T*_pc_^initial^	°C	skin	241	242	240	203	210	210
		core	248	-	245	207	-	-
Δ*h*_pc_^initial^	J/g	skin	−2.1	−1.1	−2.0	−2.7	−0.6	−0.3
		core	−1.4	-	−1.0	−3.8	-	-
*T*_M_^initial^	°C	skin	265	265	264	226	226	226
		core	266	265	265	226	227	227
Δ*h*_M_^initial^	J/g	skin	65.2	45.4	40.4	47.3	35.4	28.9
		core	71.5	50.7	42.8	48.7	36.8	32.1
*X*_C_^initial^	%	skin	24.8	24.8	25.1	32.9	31.0	29.2
		core	27.5	28.4	27.3	31.1	32.9	32.8
*T*_peak_^anneal^	°C	skin	204	212	210	194	199	203
		core	212	217	213	196	202	204
*T*_pc_^anneal^	°C	skin	243	244	242	206	213	-
		core	-	-	247	210	-	-
Δ*h*_pc_^anneal^	J/g	skin	−0.5	−0.2	−1.6	−1.4	−0.7	-
		core	-	-	−1.0	−1.8	-	-
*T*_M_^anneal^	°C	skin	265	265	264	226	225	228
		core	266	266	265	228	227	228
Δ*h*_M_^anneal^	J/g	skin	76.2	55.3	46.2	52.5	40.5	33.7
		core	82.6	58.0	48.4	53.5	41.9	36.2
*X*_C_^anneal^	%	skin	29.7	30.9	29.1	37.2	35.5	34.3
		core	32.4	32.5	31.0	36.2	37.4	36.9

**Fig. 4:**
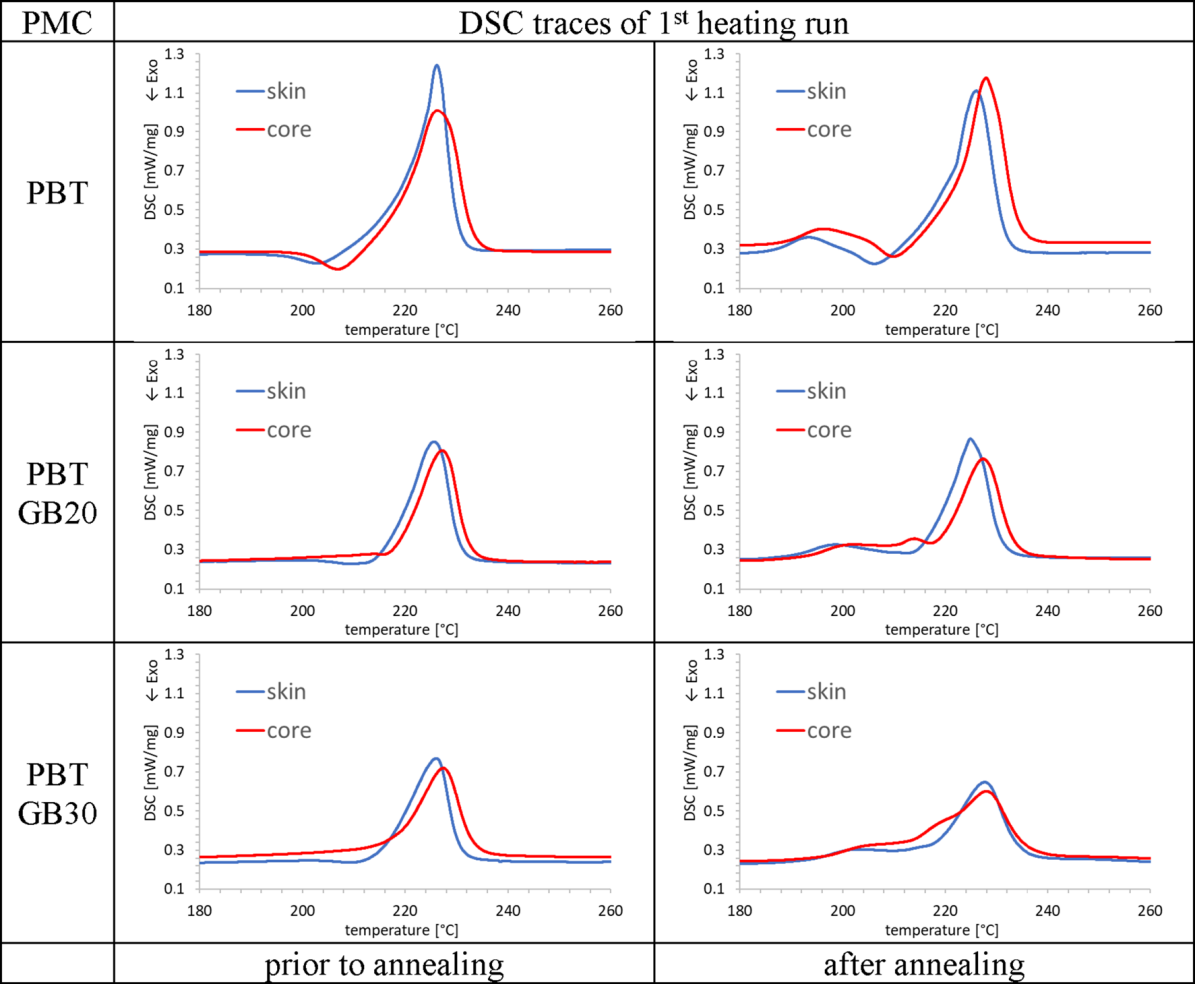
Effects of annealing for 4 h at 180 °C on DSC traces of PBT PMC, and reduction of heats of fusion with increasing GB content.

The DSC traces after annealing exhibit differences between the skin and core regions, Fig. 3. The post-crystallization peaks were less pronounced with respect to the initial state, particularly in the core regions. Although melting temperatures did not remarkably change, annealing had a large effect on the crystallinity; in the skin region it increased from 25% to 30%, and in the core region from 28% to 32%, Table 2. The DSC traces of all PBT PMC show in the initial state that GB have a nucleation effect, Fig. 4. In the skin region melting peaks occur at slightly lower temperatures compared to the core regions but this has no significant effect on the melting temperatures ranging from 226 to 227 °C. The heats of fusions are also larger for the core regions, Table 2.

The DSC traces of all annealed PBT PMC showed small peaks above 195 °C due to annealing, Fig. 4. Post-crystallization peaks are only visible for neat PBT, whereas they are shifted to higher temperatures for the core region. GB filling of PBT PMC leads to a small peak or a shoulder, respectively, in the temperature range 215 to 220 °C which is attributed to post-crystallization. Although the melting temperatures did not change, the heats of fusions increased between 3 and 5 J/g, Table 2. In the skin region, the crystallinity increased from 29% to 33%, and in the core region, from 34% to 37%. The DSC investigations showed that the crystallinity differences between skin and core were not reduced significantly due to annealing. This indicates that corresponding morphology differences remained in the samples. If annealing increases the crystallinity of PMC, their stiffness has also to be increased. Although crystallinity differences between skin and core regions were reduced, they were not levelled out. This means that the skin region has a smaller modulus than the core region. If one considers the skin core structure as a laminate it is immediately clear that flexural moduli of PMC should be slightly smaller than the corresponding longitudinal or tensile moduli.

### Methods

Prior to testing, the test bars were annealed for 4 h at 180 °C and subsequently stored for 24 h at 23 °C/50% r.h. to ensure a unified thermal history of the samples. For the IET, two sets of dumbbell specimens were cut into rectangular bars with dimension of (60 × 10 × 4) mm (set 1) and (80 × 10 × 4) mm (set 2). Samples of both lengths were tested by IET to identify geometry effects, as ASTM E1876–22^44^ recommends a length-thickness-ratio of at least 20. Only the 60 mm specimens with a length-thickness ratio of 15 were fitted to the DMA 3-point bending sample holder. Thus, after measuring them using IET, they were tested using DMA to directly compare the IET and DMA data.

The IET uses resonant frequencies of different vibration modes according to ASTM E1876-22. A specimen having a defined geometry and supports was mechanically excited to vibrations using a singular elastic impulse. The frequencies were analyzed by considering the specimen geometry and density, yielding the stiffness of the material. The IET experiments were performed with a MK7 (GrindoSonic, Leuven, Belgium), allowing for excitation of resonant frequencies in the longitudinal, flexural, or torsional mode with subsequent determination of the corresponding stiffnesses according to ASTM E1876-22. Table 3 lists the positions of the samples with the impulse points, detection points, and supports. The supports must be located at the nodal lines, thus allowing for free undisturbed vibrations.

The experiments were performed as follows:

- Positioning of the test bar according to the chosen mode.
- Hitting the test bar with a “hammer” at the impulse point on the nodal line to initiate vibrations.
- Detection of vibration frequencies of test bar at detection point.
- Data acquisition and evaluation.

**Table 3:**
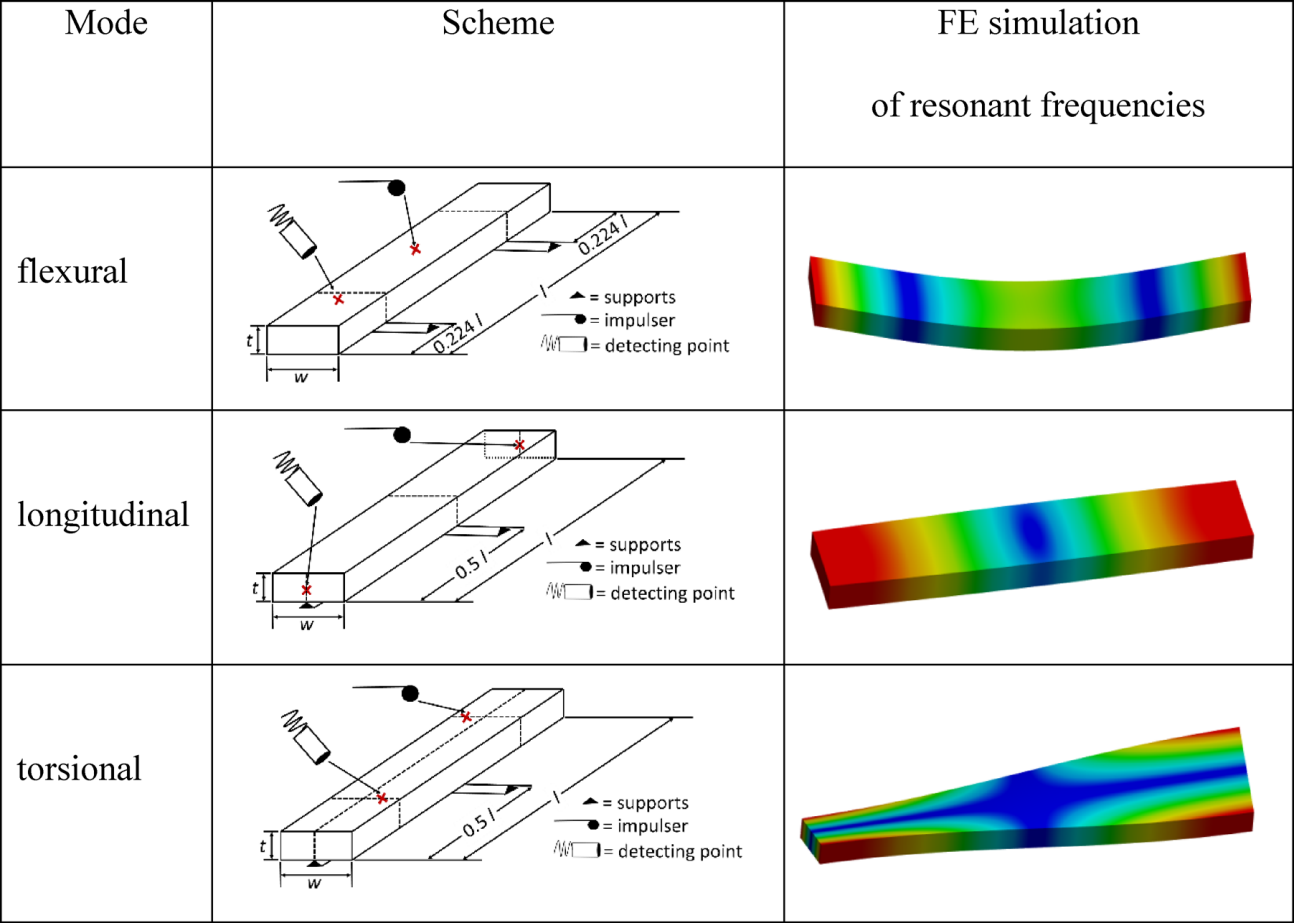
Modes of IET tests with schemes of experimental arrangement and corresponding FE analyses of the deformation behavior.

Five test bars of each grade were tested ten times. The stiffnesses of the different vibration modes were calculated using the following equations^44^:

2 $$\:\mathrm{F}\mathrm{l}\mathrm{e}\mathrm{x}\mathrm{u}\mathrm{r}\mathrm{a}\mathrm{l}\:\mathrm{m}\mathrm{o}\mathrm{d}\mathrm{e}:\:{E}_{\mathrm{f}}^{\mathrm{I}\mathrm{E}\mathrm{T}}=0.9465\left(\frac{m\:{{f}_{\mathrm{f}}}^{2}}{w}\right)\left(\frac{{l}^{3}}{{t}^{3}}\right){T}_{1}$$

with flexural modulus $\:{E}_{\mathrm{f}}^{\mathrm{I}\mathrm{E}\mathrm{T}}$, mass *m*, resonant frequency *f*_f_, length *l*, width *w* and thickness *t. T*_1_ is a form factor in flexural excitation, assuming an initial Poisson ratio *µ*:

3 $$\:{T}_{1}=1+6.585\left(1+0.0752\mu\:+0.8109{\mu\:}^{2}\right){\left(\frac{t}{l}\right)}^{2}-0.868{\left(\frac{t}{l}\right)}^{4}-\:\left(\frac{8.340\left(1+0.2023\mu\:+2.173{\mu\:}^{2}\right){\left(\frac{t}{l}\right)}^{4}}{1.000+6.338\left(1+0.1408\mu\:+1.536{\mu\:}^{2}\right){\left(\frac{t}{l}\right)}^{2}}\right)$$

4 $$\:\mathrm{L}\mathrm{o}\mathrm{n}\mathrm{g}\mathrm{i}\mathrm{t}\mathrm{u}\mathrm{d}\mathrm{i}\mathrm{n}\mathrm{a}\mathrm{l}\:\mathrm{m}\mathrm{o}\mathrm{d}\mathrm{e}:\:{E}_{\mathrm{l}}^{\mathrm{I}\mathrm{E}\mathrm{T}}=4\:m\:{{f}_{\mathrm{l}}}^{2}\left(\frac{l}{w\:t\:K}\right)$$

with the longitudinal modulus $\:{E}_{\mathrm{l}}^{\mathrm{I}\mathrm{E}\mathrm{T}}$ and longitudinal resonant frequency *f*_l_. *K* is the form factor, which depends on the Poisson ratio *µ*:

5 $$\:K=1-\left(\frac{{\pi\:}^{2}{\mu\:}^{2}}{8{l}^{2}}\:\frac{2\left({w}^{2}+{t}^{2}\right)}{3}\right)$$

6 $$\:\mathrm{T}\mathrm{o}\mathrm{r}\mathrm{s}\mathrm{i}\mathrm{o}\mathrm{n}\mathrm{a}\mathrm{l}\:\mathrm{m}\mathrm{o}\mathrm{d}\mathrm{e}:\:{G}_{\mathrm{t}}^{\mathrm{I}\mathrm{E}\mathrm{T}}=\left(\frac{4\:l\:m\:{{f}_{\mathrm{t}}}^{2}}{w\:t}\right)*\underset{C}{\underbrace{\left(\frac{B}{1\:+\:A}\right)}}$$

with shear modulus $\:{G}_{\mathrm{t}}^{\mathrm{I}\mathrm{E}\mathrm{T}}$, torsional resonant frequency *f*_t_ and form factors *B* and *A*.

7 $$\:B=\frac{\frac{w}{t}\:+\:\frac{t}{w}}{4\left(\frac{t}{w}\right)-2.52{\left(\frac{t}{w}\right)}^{2}+0.21{\left(\frac{t}{w}\right)}^{6}}$$

8 $$\:A=\frac{0.5062-08776\:\frac{w}{t}+0.3504\:{\left(\frac{w}{t}\right)}^{2}-0.0078\:{\left(\frac{w}{t}\right)}^{3}}{12.03\:\frac{w}{t}+9.892\:{\left(\frac{w}{t}\right)}^{2}}$$

Finally, the Poisson’s ratios $\:{\mu\:}_{\mathrm{f}}^{\mathrm{I}\mathrm{E}\mathrm{T}}$ and $\:{\mu\:}_{\mathrm{l}}^{\mathrm{I}\mathrm{E}\mathrm{T}}$ were determined iteratively for the flexural and longitudinal excitation modes, according to^44^.

9 $$\:{\mu\:}_{\mathrm{f}}^{\mathrm{I}\mathrm{E}\mathrm{T}}=\frac{{E}_{\mathrm{f}}^{\mathrm{I}\mathrm{E}\mathrm{T}}}{2{G}_{\mathrm{t}}^{\mathrm{I}\mathrm{E}\mathrm{T}}}-1\:{\mu\:}_{\mathrm{l}}^{\mathrm{I}\mathrm{E}\mathrm{T}}=\frac{{E}_{\mathrm{l}}^{\mathrm{I}\mathrm{E}\mathrm{T}}}{2{G}_{\mathrm{t}}^{\mathrm{I}\mathrm{E}\mathrm{T}}}-1$$

In Eq. (9), the shear modulus $\:{G}_{\mathrm{t}}^{\mathrm{I}\mathrm{E}\mathrm{T}}$ is determined directly, whereas the flexural and longitudinal moduli $\:{E}_{\mathrm{f}}^{\mathrm{I}\mathrm{E}\mathrm{T}}$ and longitudinal modulus $\:{E}_{\mathrm{l}}^{\mathrm{I}\mathrm{E}\mathrm{T}}$ depend on Poisson´s ratio (Eqs. (3) and (5)). Thus, Poisson´s ratios in Eq. (9) must be determined iteratively starting with a Poisson´s ratio taken from the literature until the absolute deviation remains smaller than 0.02, as required by ASTM E 1876−22.

Furthermore, a finite element model was built as an additional evaluation routine for impulse excitation experiments, which can be used when the geometry is not in accordance with ASTM E 1876−22. Therefore, a 3D analysis approach for determining the flexural, longitudinal, and torsional resonant frequencies was introduced to the finite element analysis (FEA) software ANSYS Workbench 2022R2. The rectangular specimens were uniformly meshed using quadratic shell elements with dimensions of 0.5 mm. Because the required elastic material parameters were unknown, an inverse parameter optimization approach was applied to determine the elastic parameters *E* and *µ* using the integrated direct optimization tool of ANSYS Workbench. The iterative parameter optimizer varied the elastic modulus and Poisson’s ratio to achieve resonant frequencies approaching those of the IET. Because the flexural and longitudinal modes are influenced by both *E* and *µ*, a multi-criteria objective function based on the torsional and flexural resonant frequencies or torsional and longitudinal resonant frequencies, respectively, was used.

DMA allows the determination of the frequency- and temperature-dependent viscoelastic behavior in terms of the complex modulus E*, storage modulus *E*´, loss modulus *E*´´, and loss factor tan *δ* according to ASTM D 4092 and DIN EN ISO 178^45^. A dynamic mechanical analyzer (DMA 242 E Artemis, Netzsch, Selb, Germany) was used to perform strain sweeps with strain amplitudes of 20, 50, 80, 110, 150, 200, and 240 μm in 3-point bending with a support distance of 50 mm at a frequency of 1 Hz and 23 °C/50% r.h. This corresponds to strain-rate amplitudes of (1.2, 3.0, 4.8, 6.6, 9.0, 12.1, 14.5)*10^− 3^ s⁻¹, respectively. The maximum dynamic force was 10.9 N with a proportionality factor of 1.1. Four test bars were measured at each strain amplitude for 5 min at 22 °C. As the measurements were performed below the glass transition temperatures of both matrix systems, low *E´´* do not lead to significant differences between E* and *E´*, thus only *E´* in 3-point bending served as comparison value further denoted $\:{E}_{\mathrm{f}}^{\prime\mathrm{D}\mathrm{M}\mathrm{A}}$.

Oscillatory torsion tests were performed using a rheometer (AR 1000, TA Instruments, New Castle, USA) equipped with a clamping device for the rectangular specimen to determine storage shear modulus $\:{G}_{\mathrm{t}}^{'\mathrm{O}\mathrm{T}}$. The initial clamping length was 45 mm. Three test bars taken out of the parallel region of dumbbell specimens with *l* = 60 mm of each PMC were measured at 1 Hz in controlled-stress (CS) mode with a maximum torque of 5 mN*m at 23 °C/50% r.h. The amplitudes obtained in CS mode correspond to a torsional strain-rate range of 5–10 s⁻¹. Subsequently, Poisson´s ratio $\:{\mu\:}_{\mathrm{f}}^{\mathrm{D}\mathrm{M}\mathrm{A}/\mathrm{O}\mathrm{T}}$ was calculated as follows:

10 $$\:{\mu\:}_{\mathrm{f}}^{\mathrm{D}\mathrm{M}\mathrm{A}/\mathrm{O}\mathrm{T}}=\frac{{E}_{\mathrm{f}}^{'\mathrm{D}\mathrm{M}\mathrm{A}}}{2{G}_{\mathrm{t}}^{'\mathrm{O}\mathrm{T}}}-1$$

where $\:{E}_{\mathrm{f}}^{'\mathrm{D}\mathrm{M}\mathrm{A}}$ represents the flexural storage modulus obtained from 3-point bending measurements in the DMA, and $\:{G}_{\mathrm{t}}^{'\mathrm{O}\mathrm{T}}$ was evaluated from the oscillatory torsion measurements.

Tensile tests provide stress–strain curves from which relevant static mechanical properties such as static stiffness, strength, and elongation at break are elucidated according to DIN EN ISO 527-1^45^. Furthermore, Poisson´s ratio can be obtained by measuring the deformation behavior parallel and perpendicular to the load direction. Tensile tests were performed at 23 °C/50% r.h using a universal testing machine (Zwick Z010, Zwick/Roell, Ulm, Germany) equipped with a 10 kN load cell (resolution: 0.12%) and laser extensometer (Zwick/Roell laserXtens extensometer 2–220 HP; resolution: 0.1 μm) using specimen geometry type 1 A. The initial clamping length was set as 80 mm. Simultaneous determination of the time-dependent length and width was required to set the initial length and width of the laser to 22 and 9 mm, respectively. Cyclic deformation steered tensile tests were performed in the linear elastic region between 100 and 1000 N at a displacement rate of 1 mm/min, corresponding to a strain rate of 7.6 * 10^− 4^ s⁻¹. Four test bars of each PMC were subjected to six cycles and evaluated using the slope during unloading of the last five cycles with respect to Young´s modulus $\:{E}_{\mathrm{l}}^{\mathrm{T}\mathrm{T}}$ and Poisson’s ratio $\:{\mu\:}_{\mathrm{l}}^{\mathrm{T}\mathrm{T}}$:

11 $$\:{E}_{\mathrm{l}}^{\mathrm{T}\mathrm{T}}=\:\frac{1}{n-1}\:{\sum\:}_{i=2}^{n-1}{E}_{i}$$

12 $$\:{\mu\:}_{\mathrm{l}}^{\mathrm{T}\mathrm{T}}=\frac{1}{n-1}\:\frac{{l}_{0}}{{w}_{0}}\:{\sum\:}_{i=2}^{n-1}\frac{{\varDelta\:w}_{i}}{{\varDelta\:l}_{i}}$$

where *n* is the number of cycles, Δ*w* stands for change of width, Δ*l* represents change in length, initial length *l*_0_ and width *w*_0_. Because of the smooth surface of the PBT-based PMC, the test bars were sprayed with a speckle spray to enhance the laser signal, allowing for more precise measurements of Δ*l* and Δ*w*.

## Results and discussion

The following findings and discussion refer to the determination of elastic constants in the linear regime of polymers and composites, considering various methods and the factors affecting the results for defined conditions (23 °C/50% r.h.).

### *Comparison of moduli determined by IET according* to *ASTM E1876-22 and FEA*

The measured IET resonant frequencies of both the 60 mm and 80 mm test bars (Table 4) reveal that a higher filler volume content *v*_F_ increases the corresponding resonant frequency of each vibration mode, which is attributed to the stiffening effect of the glass beads. Furthermore, the resonant frequency depends mainly on the specimen´s length, which results in lower resonant frequencies for the longer specimen. It is shown that the differences between measured and simulated frequencies is smaller than 1% for each vibration mode and material, confirming the experimental evaluation according to ASTM E1876-22 for the moduli $\:{E}_{\mathrm{f}}^{\mathrm{I}\mathrm{E}\mathrm{T}}$, $\:{E}_{\mathrm{l}}^{\mathrm{I}\mathrm{E}\mathrm{T}}$, and $\:{G}_{\mathrm{t}}^{\mathrm{I}\mathrm{E}\mathrm{T}}$ in flexural, longitudinal, and torsional vibration modes, respectively.

Neat polymers exhibit at least two-fold larger relative errors than the PMC with the highest GB content. This can be attributed to the higher damping behavior of unfilled polymers. In case of the stiffnesses, $\:{G}_{\mathrm{c}\mathrm{a}\mathrm{l}\mathrm{c}}^{\mathrm{I}\mathrm{E}\mathrm{T}}$ were determined directly using Eq. (6), $\:{E}_{\mathrm{c}\mathrm{a}\mathrm{l}\mathrm{c}}^{\mathrm{I}\mathrm{E}\mathrm{T}}$ were determined iteratively using Eqs. (2), (4) and (9), Table 4. However, with respect to the accuracy of the measurements, test bars having a length-thickness-ratio of 15 already provide reliable stiffnesses.

 The evaluation of longitudinal $\:{E}_{\mathrm{c}\mathrm{a}\mathrm{l}\mathrm{c}}^{\mathrm{I}\mathrm{E}\mathrm{T}}$ yields larger Young’s moduli (4 to 8% for PA66 and 2 to 4% for PBT) than the evaluation of flexural $\:{E}_{\mathrm{c}\mathrm{a}\mathrm{l}\mathrm{c}}^{\mathrm{I}\mathrm{E}\mathrm{T}}$. These small stiffness discrepancies can be attributed firstly to structure inhomogeneities due to the skin core structure with reduced crystallinities and slightly reduced GB concentrations in the skin region compared to the core region caused by the flow conditions during injection molding, Sect. 2.1, and secondly to 5 to 10 higher resonant frequencies of the longitudinal mode increasing the moduli due to the viscoelastic behavior of the matrix. The moduli discrepancies are less pronounced for PBT composites because of their higher crystallization rate and higher crystallinity. The applied stress in bending excitation leads to larger strains in the skin region and thus to lower moduli compared to the longitudinal excitation, where skin and core experience the same strain.

The FEA optimization procedure yields values of $\:{E}_{\mathrm{F}\mathrm{E}\mathrm{A}}^{\mathrm{I}\mathrm{E}\mathrm{T}}$ and $\:{G}_{\mathrm{F}\mathrm{E}\mathrm{A}}^{\mathrm{I}\mathrm{E}\mathrm{T}}$which are very similar to those of $\:{E}_{\mathrm{c}\mathrm{a}\mathrm{l}\mathrm{c}}^{\mathrm{I}\mathrm{E}\mathrm{T}}$ and $\:{G}_{\mathrm{c}\mathrm{a}\mathrm{l}\mathrm{c}}^{\mathrm{I}\mathrm{E}\mathrm{T}}$ for all PMC and modes. Thus, FEA can be considered as a second possibility for evaluating IET data. It should be noted that in Table 4, $\:{E}_{\mathrm{F}\mathrm{E}\mathrm{A}}^{\mathrm{I}\mathrm{E}\mathrm{T}}$ and $\:{G}_{\mathrm{F}\mathrm{E}\mathrm{A}}^{\mathrm{I}\mathrm{E}\mathrm{T}}$ are not denoted with STD, as the FEA optimization procedures were executed using the mean values of resonant frequency and density for constant test bar geometries to simulate the stiffnesses.

**Table 4:** Resonant frequencies and moduli obtained from IET and FEA of polymer matrix composites (PMC) with different test bar lengths *l*.

	PMC		l = 60 mm			l = 80 mm		
Mode	Matrix	*w*_F_	Resonant frequency	$\:{E}_{\mathrm{c}\mathrm{a}\mathrm{l}\mathrm{c}}^{\mathrm{I}\mathrm{E}\mathrm{T}}$	$\:{E}_{\mathrm{F}\mathrm{E}\mathrm{A}}^{\mathrm{I}\mathrm{E}\mathrm{T}}$	Resonant frequency	$\:{E}_{\mathrm{c}\mathrm{a}\mathrm{l}\mathrm{c}}^{\mathrm{I}\mathrm{E}\mathrm{T}}$	$\:{E}_{\mathrm{F}\mathrm{E}\mathrm{A}}^{\mathrm{I}\mathrm{E}\mathrm{T}}$
		%	Hz	MPa	MPa	Hz	MPa	MPa
flexural	PA66	0	1861 ± 36	3261 ± 18	3219	1082 ± 61	3213 ± 37	3214
		30	2199 ± 14	4950 ± 55	4920	1224 ± 8	4944 ± 52	4927
		40	2335 ± 12	5736 ± 42	5723	1291 ± 4	5673 ± 53	5691
	PBT	0	1670 ± 19	2795 ± 19	2812	897 ± 23	2775 ± 33	2759
		20	1917 ± 28	3865 ± 25	3839	1063 ± 4	3712 ± 27	3718
		30	2005 ± 11	4507 ± 15	4498	1132 ± 6	4504 ± 25	4483
longitudinal	PA66	0	14,124 ± 131	3386 ± 11	3353	10,721 ± 30	3343 ± 15	3343
		30	16,642 ± 56	5322 ± 23	5362	12,358 ± 42	5293 ± 18	5249
		40	17,186 ± 48	5997 ± 16	5972	12,712 ± 19	5927 ± 23	5928
	PBT	0	12,386 ± 69	2880 ± 10	2900	9036 ± 17	2855 ± 13	2825
		20	14,012 ± 103	3930 ± 6	3909	10,399 ± 23	3852 ± 11	3859
		30	14,792 ± 53	4682 ± 13	4686	11,074 ± 29	4659 ± 10	4659
Mode	Matrix	*w*_F_	Resonant frequency	$\:{G}_{\mathrm{c}\mathrm{a}\mathrm{l}\mathrm{c}}^{\mathrm{I}\mathrm{E}\mathrm{T}}$	$\:{G}_{\mathrm{F}\mathrm{E}\mathrm{A}}^{\mathrm{I}\mathrm{E}\mathrm{T}}$	Resonant frequency	$\:{G}_{\mathrm{c}\mathrm{a}\mathrm{l}\mathrm{c}}^{\mathrm{I}\mathrm{E}\mathrm{T}}$	$\:{G}_{\mathrm{F}\mathrm{E}\mathrm{A}}^{\mathrm{I}\mathrm{E}\mathrm{T}}$
		%	Hz	MPa	MPa	Hz	MPa	MPa
torsional	PA66	0	5328 ± 61	1139 ± 12	1145	4048 ± 14	1132 ± 9	1138
		30	6270 ± 24	1791 ± 1	1799	4651 ± 14	1780 ± 15	1798
		40	6631 ± 14	2113 ± 16	2101	4901 ± 9	2095 ± 20	2100
	PBT	0	4701 ± 28	978 ± 5	979	3429 ± 45	972 ± 7	977
		20	5373 ± 41	1378 ± 5	1380	3986 ± 9	1344 ± 8	1346
		30	5705 ± 20	1640 ± 4	1637	4277 ± 11	1639 ± 6	1636

* ± represents the standard deviations of five specimens.

The resonant frequencies of IET exceed the frequencies of DMA 3 or 4 orders of magnitude. Thus, one has to expect larger moduli for IET because of glass transitions shifted to higher temperatures.

### *Comparison of flexural moduli E*_f_*determined by IET and DMA in 3-point bending mode*

**Fig. 5:**
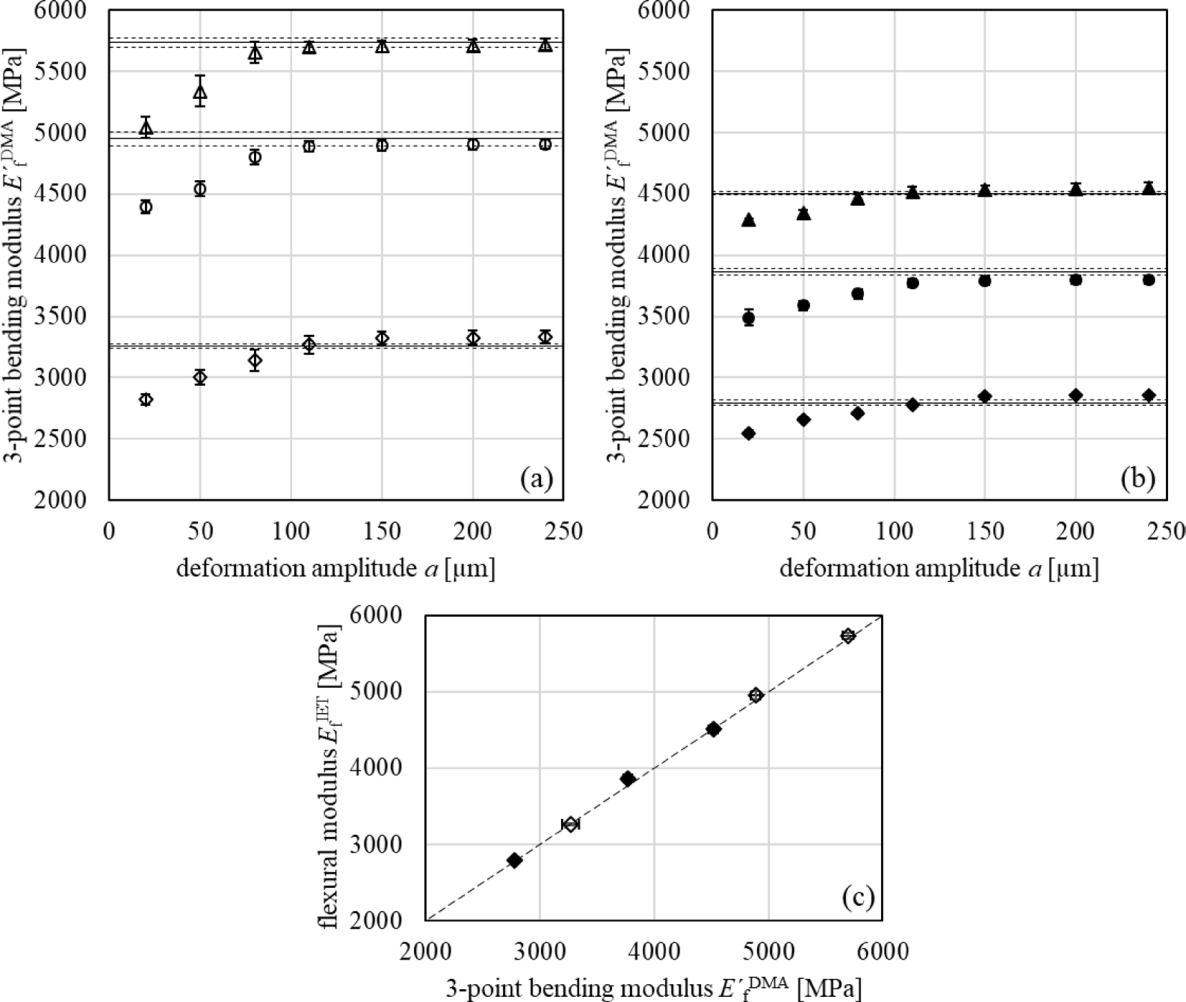
Deformation amplitude *a* dependent Young’s moduli $\:{E}_{\mathrm{f}}^{'\mathrm{D}\mathrm{M}\mathrm{A}}$ in 3-point bending mode (symbols) of PMC with PA66 matrix (**a**) and PBT matrix (**b**); the flexural $\:{E}_{\mathrm{f}}^{\mathrm{I}\mathrm{E}\mathrm{T}}$ (solid lines) with STD (dashed lines) are included for comparison. (**c**) depicts the correlation between both moduli of PMC´s of PA66 (empty symbols) and PBT (full symbols).

The DIN EN ISO 178 permits strains of the outer fiber deformations ranging from 0.05 to 0.25% to determine flexural properties of plastics. Therefore, strain amplitude sweeps were performed by the DMA with stepwise increasing deformation amplitudes Δ*x*_0_ from 20 to 240 μm, corresponding to strains ranging from 0.02 to 0.23%. Again, increasing filler volume contents *v*_F_ from neat PA66 to reinforced PA66 GB30 and PA66 GB40 lead to an increase in stiffness $\:{E}_{\mathrm{f}}^{'\mathrm{D}\mathrm{M}\mathrm{A}}$ by 47% and 72%, respectively, and from neat PBT to reinforced PBT GB20 and PBT GB30 to an increase in stiffness $\:{E}_{\mathrm{f}}^{'\mathrm{D}\mathrm{M}\mathrm{A}}$ by 33% and 59%, respectively, **Appendix A2**. The increase of the deformation amplitudes led to increasing stiffnesses $\:{E}_{\mathrm{f}}^{'\mathrm{D}\mathrm{M}\mathrm{A}}$ for deformation amplitudes up to 110 μm independently of the filler content, Fig. 5.

This stiffening behavior cannot arise from the viscoelastic behavior of the PMC as this would lead to softening. It has to originate from a change of the experimental boundary conditions for small forces due to friction and sliding of the test bars on the bearing, partial contact of the test bar to the bearing because of a little warpage, and a minor indent of the excitation rod prior to bending. Only for deformation amplitudes exceeding 110 μm the method intrinsic errors were overcome and the applied force led to a defined contact of test bar and bearing resulting in constant flexural storage moduli $\:{E}_{\mathrm{f}}^{'\mathrm{D}\mathrm{M}\mathrm{A}}$ of all PMC, Fig. 5**(a-b)**. This assumption was also confirmed by the damping behavior tan *δ*. Only exceeding the threshold amplitude lead to a constant value due to defined contact conditions. With respect to the STD, the $\:{E}_{\mathrm{f}}^{'\mathrm{D}\mathrm{M}\mathrm{A}}$ is identical to the flexural $\:{E}_{\mathrm{f}}^{\mathrm{I}\mathrm{E}\mathrm{T}}$ for the 60 mm test bars, Fig. 5**(c)**. Comparing $\:{E}_{\mathrm{f}}^{'\mathrm{D}\mathrm{M}\mathrm{A}}$ to $\:{E}_{\mathrm{f}}^{\mathrm{I}\mathrm{E}\mathrm{T}}$ is a sophisticated issue for three reasons: (1) the resonant frequencies of IET measurements are three to four orders of magnitude larger than those in a DMA measurement. In the case of PMC, $\:{E}_{\mathrm{f}}^{\mathrm{I}\mathrm{E}\mathrm{T}}$ is expected to exceed $\:{E}_{\mathrm{f}}^{'\mathrm{D}\mathrm{M}\mathrm{A}}$ because of the time-temperature superposition principle; (2) deformation amplitudes in an IET measurement are smaller than those in a DMA measurement; (3) free vibrations occur in IET measurements, whereas in DMA measurements forced deformation is applied.

### *Comparison of longitudinal moduli E*_l_*determined by IET and TT*

**Fig. 6:**
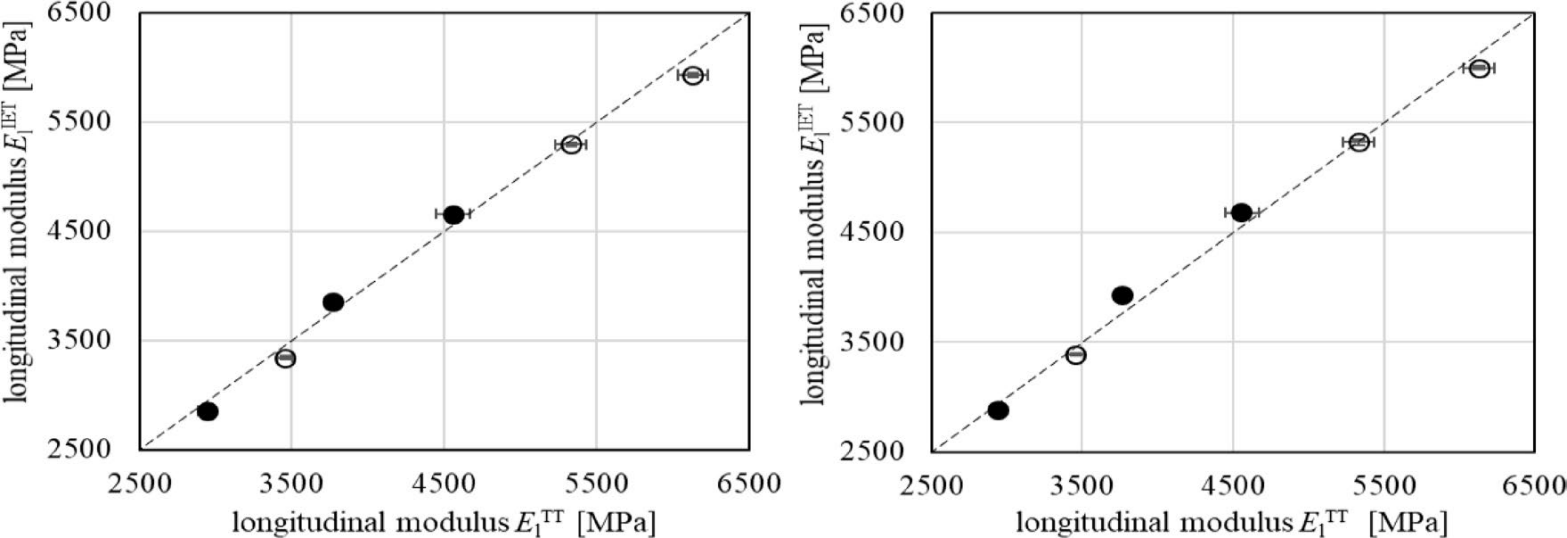
Longitudinal moduli with standard deviations obtained from IET and TT for composites with PA66 (empty symbols) and PBT (full symbols) matrices having sample length *l* = 60 mm (**a**) and *l* = 80 mm (**b**).

The Young´s moduli $\:{E}_{\mathrm{l}}^{\mathrm{T}\mathrm{T}}$ were evaluated using the unloading sections of the *σ* – *ε*-curves. Once more, moduli increase monotonously with increasing filler volume content *v*_F_ due to the stiffening effect of GB, **Appendix A3**. The comparison with $\:{E}_{\mathrm{l}}^{\mathrm{I}\mathrm{E}\mathrm{T}}$ shows that the stiffnesses yielded by both methods coincide with the increasing moduli and STD. However, considering the STD a larger error susceptibility occurs for tensile testing, Fig. 6. Although cyclic deformation–controlled TT can avoid intrinsic acceleration errors and maintain constant strain rates during testing, their higher susceptibility to error can be attributed to the weak interaction between the laser and the polymer surface.

### Comparison of torsional modulus G determined by IET and OT

**Fig. 7:**
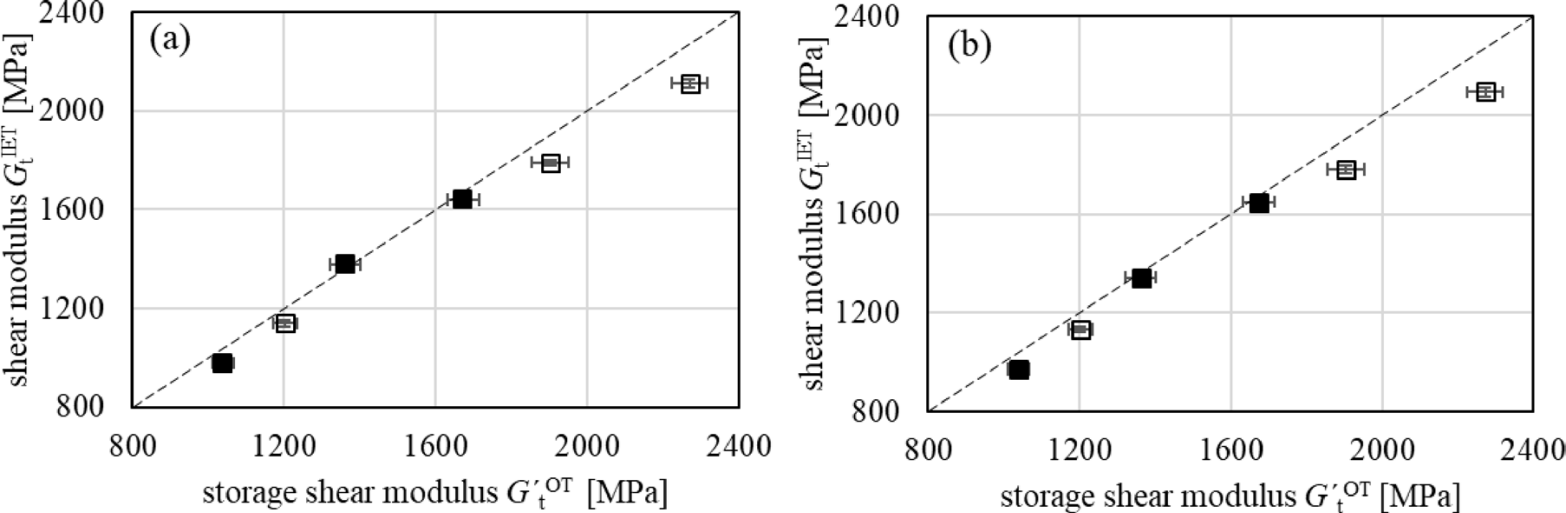
Torsion moduli obtained with OT ($\:{G}_{\mathrm{t}}^{'\mathrm{O}\mathrm{T}})$ and IET ($\:{G}_{\mathrm{t}}^{\mathrm{I}\mathrm{E}\mathrm{T}})$ with STD for composites with PA66 (empty symbols) and PBT (full symbols) matrices having sample length *l* = 60 mm (a) and *l* = 80 mm (b).

The shear moduli $\:{G}_{\mathrm{t}}^{'\mathrm{O}\mathrm{T}}$ were evaluated for oscillatory torsional loading at a frequency of 1 Hz and show as well increasing moduli with increasing GB content *v*_F_. For the PA66 composites, approximately 50 MPa larger $\:{G}_{\mathrm{t}}^{\mathrm{O}\mathrm{T}}$ was found compared to $\:{G}_{\mathrm{t}}^{\mathrm{I}\mathrm{E}\mathrm{T}}$. For PBT composites, slightly higher means of $\:{G}_{\mathrm{t}}^{'\mathrm{O}\mathrm{T}}$ were observed but coinciding within the STD, **Appendix A4**. This might be compensated for PBT composites with respect to the aforementioned homogenous morphology and, thus, resulting in smaller differences. Due to higher deformation rates in the OT measurements (5–10 s⁻¹) compared to TT (7.6 × 10⁻⁴ s⁻¹) and DMA (14.4 × 10⁻³ s⁻¹), higher moduli can be expected for OT for viscoelastic reasons. The oscillatory torsion test appears to be slightly more erroneous than the IET test, as clamping issues affect torque transmission and thus result in a STD., Fig. 7.

#### Comparison of Poisson´s ratio µ: IET, TT, DMA and OT

Poisson´s ratios $\:{\mu\:}_{\mathrm{f}}^{\mathrm{D}\mathrm{M}\mathrm{A}/\mathrm{O}\mathrm{T}}$ were determined using Eq. (10), and $\:{\mu\:}_{\mathrm{f}}^{\mathrm{I}\mathrm{E}\mathrm{T}}$ via an iterative approach according to^44^. Both approaches require isotropic material behavior, whereas $\:{\mu\:}_{\mathrm{l}}^{\mathrm{T}\mathrm{T}}$ is determined by simultaneously measuring width and length changes during tensile tests. The Poisson’s ratios decrease for both PMC types with increasing GB content as glass has smaller Poisson’s ratios ranging between 0.18 and 0.3 compared to semi-crystalline thermoplastics ranging between 0.4 and 0.45^46^.

The IET and TT methods yield Poisson’s ratios coinciding with respect to STD, Table 5, but the IET method shows that the STD is one-third or one-half that of TT. The Poisson´s ratios determined using the DMA and OT methods exhibit a similar GB content dependency as the IET and TT methods. They underestimated the Poisson´s ratios for the PMC of PA66 and overestimated them for the PMC of PBT but with higher standard deviations. These under- and over-estimations result from the assumption of linear correlation of the dynamic methods of DMA and OT (Eq. (10)), thus depending on the error susceptibility of both methods, which is further influenced by the inhomogeneous structure of the samples. In contrast, $\:{\mu\:}_{\mathrm{f}}^{\mathrm{I}\mathrm{E}\mathrm{T}}$ and $\:{\mu\:}_{\mathrm{l}}^{\mathrm{T}\mathrm{T}}$ are only limited by their own method accuracies; therefore, a better comparison of Poisson´s ratios are achieved if influenced only by one method.

**Table 5:** Poisson’s ratios of composites with PA66 and PBT matrices obtained from OT/DMA, IET and TT data.

Matrix	w_F_	$\:{\mu\:}_{\mathrm{f}}^{\mathrm{D}\mathrm{M}\mathrm{A}/\mathrm{O}\mathrm{T}}$	$\:{\mu\:}_{\mathrm{f}}^{\mathrm{I}\mathrm{E}\mathrm{T}}$ l = 60 mm	$\:{\mu\:}_{\mathrm{f}}^{\mathrm{I}\mathrm{E}\mathrm{T}}$ l = 80 mm	$\:{\mu\:}_{\mathrm{l}}^{\mathrm{T}\mathrm{T}}$
	%				
PA66	0	0.38 ± 0.01	0.43 ± 0.01	0.42 ± 0.01	0.44 ± 0.03
	30	0.32 ± 0.03	0.38 ± 0.01	0.39 ± 0.01	0.40 ± 0.02
	40	0.31 ± 0.06	0.36 ± 0.01	0.35 ± 0.01	0.35 ± 0.02
PBT	0	0.45 ± 0.02	0.43 ± 0.01	0.43 ± 0.01	0.42 ± 0.03
	20	0.45 ± 0.06	0.40 ± 0.01	0.38 ± 0.01	0.38 ± 0.04
	30	0.43 ± 0.04	0.37 ± 0.01	0.37 ± 0.01	0.36 ± 0.01

From the literature survey, it is clear that the mechanical behavior mainly depends on the excitation direction owing to systematic factors such as flow-induced orientations, formation of skin core morphologies and agglomerations of filler particles, and filler volume content. These inhomogeneities have been investigated for many composite systems, e.g^6,7,10–12^. found $\:{E}_{\mathrm{l}}$ > $\:{E}_{\mathrm{f}}$, whereas^8,9,13,14^ observed $\:{E}_{\mathrm{l}}$ < $\:{E}_{\mathrm{f}}$. No clear tendency was shown for methods comparison, including tensile test, DMA, or oscillatory torsion, and only two studies^29,30^ implemented IET.

The IET method is currently used mainly for stiff materials, such as metals, ceramics, and their composites, as it can provide mechanical data in a reliable, fast, and simple manner. Therefore, it would be interesting to extend the IET method to polymers and PMC. The properties resulting from the IET method ($\:{E}_{\mathrm{f}}^{\mathrm{I}\mathrm{E}\mathrm{T}}$, $\:{E}_{\mathrm{l}}^{\mathrm{I}\mathrm{E}\mathrm{T}}$, $\:{G}_{\mathrm{t}}^{\mathrm{I}\mathrm{E}\mathrm{T}}$, and $\:{\mu\:}_{\mathrm{f}}^{\mathrm{I}\mathrm{E}\mathrm{T}})$ were correlated with the properties measured by tensile tests, DMA, and oscillatory torsion using the correlation coefficient *r* according to Pearson. Furthermore, the significance factor *p* was calculated alongside the Pearson coefficient, where only statistical significance is given for *p*-values < 0.05.

Correlation coefficients according to Pearson show a linear relationship for all method comparisons, especially pronounced for the elastic moduli, Table 6. However, Poisson´s ratios *µ* still exhibit linear correlations, but with higher error susceptibilities for quasi-static TT and dynamic DMA/OT compared with IET, but the correlation is not statistically significant. Furthermore, the definition of the Poisson´s ratio *µ* in terms of elasticity theory requires isotropy and homogeneity. However, morphological and thermal investigations exhibited an inhomogeneous skin core structure that is slightly anisotropic. This may explain these deviations.

**Table 6:** Correlation of elastic properties of polymer matrix composites (PMC) obtained with IET to those from DMA, TT and OT methods.

Matrix	IET property	*r*	*p*
PA66	$\:{E}_{\mathrm{f}}^{\mathrm{I}\mathrm{E}\mathrm{T}}$.vs $\:{E}_{\mathrm{f}}^{'\mathrm{D}\mathrm{M}\mathrm{A}}$	1.00	0.01
	$\:{E}_{\mathrm{l}}^{\mathrm{I}\mathrm{E}\mathrm{T}}$ vs. $\:{E}_{\mathrm{l}}^{\mathrm{T}\mathrm{T}}$	1.00	0.03
	$\:{\mu\:}_{\mathrm{f}}^{\mathrm{I}\mathrm{E}\mathrm{T}}\:\mathrm{v}\mathrm{s}\:{\mu\:}_{\mathrm{f}}^{\mathrm{D}\mathrm{M}\mathrm{A}/\mathrm{O}\mathrm{T}}\:$	0.99	0.08
	$\:{\mu\:}_{\mathrm{f}}^{\mathrm{I}\mathrm{E}\mathrm{T}}$ vs. $\:{\mu\:}_{\mathrm{l}}^{\mathrm{T}\mathrm{T}}$	0.96	0.18
	$\:{G}_{\mathrm{t}}^{\mathrm{I}\mathrm{E}\mathrm{T}}$ vs. $\:{G}_{\mathrm{t}}^{'\mathrm{O}\mathrm{T}}$	1.00	0.00
PBT	$\:{E}_{\mathrm{f}}^{\mathrm{I}\mathrm{E}\mathrm{T}}$. vs. $\:{E}_{\mathrm{f}}^{'\mathrm{D}\mathrm{M}\mathrm{A}}$	1.00	0.04
	$\:{E}_{\mathrm{l}}^{\mathrm{I}\mathrm{E}\mathrm{T}}$ vs. $\:{E}_{\mathrm{l}}^{\mathrm{T}\mathrm{T}}$	1.00	0.05
	$\:{\mu\:}_{\mathrm{f}}^{\mathrm{I}\mathrm{E}\mathrm{T}}\:\mathrm{v}\mathrm{s}\:{\mu\:}_{\mathrm{f}}^{\mathrm{D}\mathrm{M}\mathrm{A}/\mathrm{O}\mathrm{T}}\:$	0.90	0.29
	$\:{\mu\:}_{\mathrm{f}}^{\mathrm{I}\mathrm{E}\mathrm{T}}$ vs. $\:{\mu\:}_{\mathrm{l}}^{\mathrm{T}\mathrm{T}}$	0.99	0.09
	$\:{G}_{\mathrm{t}}^{\mathrm{I}\mathrm{E}\mathrm{T}}$ vs. $\:{G}_{\mathrm{t}}^{'\mathrm{O}\mathrm{T}}$	1.00	0.03

Discrepancies between IET, DMA, TT, and OT can originate from various factors: morphological inhomogeneities, different loading modes, varying strain rates and frequency ranges as well as distinct boundary conditions. Although polymers and composites exhibit pronounced time-dependent behavior, the discrepancies identified in the present investigations are less a consequence of viscoelastic material effects, and more a result of morphological differences revealed by the respective loading modes. The determined STD of all IET excitations were only one-third of the maximum errors of IET, DMA, and TT being identical with respect to STD, Table 7. The maximum error estimation of all methods was performed by calculating the partial differentials of the considered quantity in the following way:

13 $$\:\varDelta\:y=\left|\frac{\partial\:y}{\partial\:{x}_{1}}\right|*\varDelta\:{x}_{1}+\left|\frac{\partial\:y}{\partial\:{x}_{2}}\right|*\varDelta\:{x}_{2}+\dots\:$$

Exemplified by the flexural modulus

2 $$\:{E}_{\mathrm{f}}^{\mathrm{I}\mathrm{E}\mathrm{T}}=0.9465\left(\frac{m\:{{f}_{\mathrm{f}}}^{2}}{w}\right)\left(\frac{{l}^{3}}{{t}^{3}}\right){T}_{1}$$

the maximum error is given by

14 $$\:\varDelta\:{E}_{\mathrm{f}}^{\mathrm{I}\mathrm{E}\mathrm{T}}=0.9465\left\{\left|\left(\frac{{{f}_{\mathrm{f}}}^{2}}{w}\right)\left(\frac{{l}^{3}}{{t}^{3}}\right){T}_{1}\right|\:\varDelta\:m\:+\:\left|2\left(\frac{m\:{f}_{\mathrm{f}}}{w}\right)\left(\frac{{l}^{3}}{{t}^{3}}\right){T}_{1}\right|\:\varDelta\:{f}_{f}+\dots\:\right\}\:$$

and appropriate expanding leads to

15 $$\:\varDelta\:{E}_{\mathrm{f}}^{\mathrm{I}\mathrm{E}\mathrm{T}}={E}_{\mathrm{f}}^{\mathrm{I}\mathrm{E}\mathrm{T}}\:\left\{\left|\frac{\varDelta\:m}{m}\right|\:+\:2\left|\frac{\varDelta\:{f}_{\mathrm{f}}}{{f}_{\mathrm{f}}}\right|+\left|-\frac{\varDelta\:w}{w}\right|+3\left|\frac{\varDelta\:l}{l}\right|+\:3\left|-\frac{\varDelta\:t}{t}\right|+\left|\frac{\varDelta\:{T}_{1}}{{T}_{1}}\right|\right\}\:\:\:$$

**Table 7:** Absolute and relative errors of elastic moduli obtained with IET, DMA, TT and OT of PA66 and PBT PMC.

Matrix	*w*_F_	$\:{\varDelta\:E}_{\mathrm{f}}^{\mathrm{I}\mathrm{E}\mathrm{T}}$	$\:{\varDelta\:E}_{\mathrm{l}}^{\mathrm{I}\mathrm{E}\mathrm{T}}$	$\:\varDelta\:{G}_{\mathrm{t}}^{\mathrm{I}\mathrm{E}\mathrm{T}}$	$\:{\varDelta\:E}_{\mathrm{f}}^{'\mathrm{D}\mathrm{M}\mathrm{A}}$	$\:{\varDelta\:E}_{\mathrm{l}}^{\mathrm{T}\mathrm{T}}$	$\:{\varDelta\:G}_{\mathrm{t}}^{'\mathrm{O}\mathrm{T}}$
	%	MPa/%					
PA66	0	34/1.0	16/0.5	21/1.9	88/2.6	65/1.9	33/2.8
	30	52/1.0	26/0.5	33/1.9	130/2.7	100/1.9	58/3.2
	40	59/1.0	29/0.5	39/1.9	155/2.7	115/1.9	73/3.4
PBT	0	35/1.0	14/0.5	18/1.9	75/2.6	55/1.9	25/2.6
	20	39/1.1	19/0.5	25/1.9	105/2.7	71/1.9	37/2.8
	30	47/1.1	23/0.5	31/1.9	121/2.6	86/1.9	47/3.0

This procedure was adapted for all methods to calculate the maximum error. Only OT partially exhibited higher variations in multiple determinations than in the maximum error estimation. Considering the maximum errors of the four methods, the IET can be considered as the most accurate measurement technique.

## Conclusion

This study evaluated the impulse excitation technique (IET) for determining elastic constants of glass bead reinforced PA66 and PBT and compared the results with dynamic mechanical analysis (DMA), tensile testing (TT) and oscillatory torsion (OT). The main objective was to assess whether IET can provide reliable elastic moduli and Poisson’s ratios for thermoplastic particulate composites under small, elastic strains and how its results relate to established mechanical test methods.

Key findings are:

- IET provided moduli that were comparable to those obtained by DMA, TT and OT, and enabled direct access to Poisson’s ratio from the same non-destructive measurements, which is advantageous for engineering design and numerical modelling of composite structures.
- In flexural excitation, matching moduli within the measurement accuracies were obtained between DMA and IET only when the dynamic threshold amplitudes in DMA exceeded 110 μm in amplitude sweep mode, indicating that sufficiently small strain amplitudes are required to ensure purely elastic behavior in DMA for these materials.
- Longitudinal IET moduli compared to TT, and torsional IET moduli compared to OT, were in good agreement but showed higher error susceptibility for TT and OT due to clamping conditions and geometric sensitivities.
- Slight differences between flexural and longitudinal moduli were observed and can be attributed to the skin–core structure of the injection molded specimens, with varying crystallinities and filler concentrations across the cross section.

Overall, the results show that IET is suitable for determining elastic constants of thermoplastic glass bead composites in the linear regime and that it delivers consistent values across different excitation modes when the testing conditions and specimen morphology are considered. The present work is restricted to small strains, and temperatures below glass transition of thermoplastic matrices (PA66 and PBT) with spherical glass bead fillers, processed under conditions that minimize defects, and does not address environmental influences, aging, damage evolution or complex geometries. Future studies will extend the present approach to other matrix–filler systems and application-oriented conditions, and will combine IET with complementary techniques such as digital image correlation, X-ray computed tomography, advanced SEM-based statistical image analysis, and additional mechanical tests (e.g. flexural strength and impact) to further quantify anisotropy, defect sensitivity, and long-term behavior of polymer composites.

## Supplementary Information

Below is the link to the electronic supplementary material.

Supplementary Material 1

## Data Availability

https://doi.org/10.5281/zenodo.17799803.
