# Supplementary material for: Towards reliable elastic characterization of glass bead reinforced thermoplastic composites using impulse excitation and conventional testing
Source: Sci Rep. 2026 Jan 22;16:5979. doi: 10.1038/s41598-026-36346-z (PMC12901990; doi:10.1038/s41598-026-36346-z)
Supplement: Supplementary file 1 — Supplementary Material 1 [file 41598_2026_36346_MOESM1_ESM.docx]

**Appendix**

**Table A1.** Cross-sectional GB distribution for both PA66 and PBT composites by counting the GB in steps with a step-width area of (200x2000) µm.

| Distance skin layer | PA66 GB30 | PA66 GB40 | PBT GB20 | PBT GB30 |
| --- | --- | --- | --- | --- |
| µm | - | - | - | - |
| 100 | 71 | 127 | 92 | 106 |
| 300 | 84 | 159 | 101 | 119 |
| 500 | 92 | 169 | 109 | 126 |
| 700 | 88 | 159 | 101 | 125 |
| 900 | 104 | 161 | 98 | 137 |
| 1100 | 103 | 173 | 101 | 116 |

**Table A2.** Comparison of ${E´}_{f}^{\mathrm{DMA}}$ (deformation amplitude = 110 µm) to $E_{f}^{\mathrm{IET}}$ and $E_{l}^{\mathrm{IET}}$ with standard deviations and coefficients of variation CV for composites with PA66 and PBT matrices.

| Matrix | *w*_F_ | ${E´}_{f}^{\mathrm{DMA}}$ / CV  3-point bending | $E_{f}^{\mathrm{IET}}$ / CV  flexural | $E_{l}^{\mathrm{IET}}$ / CV  longitudinal |
| --- | --- | --- | --- | --- |
|  | % | MPa / % | MPa / % | MPa / % |
| PA66 | 0 | 3270 ± 75 / 2.29 | 3261 ± 18 / 0.55 | 3386 ± 11 / 0.32 |
|  | 30 | 4890 ± 41 / 0.84 | 4950 ± 55 / 1.11 | 5322 ± 23 / 0.43 |
|  | 40 | 5700 ± 46 / 0.81 | 5736 ± 42 / 0.73 | 5997 ± 16 / 0.27 |
| PBT | 0 | 2781 ± 9 / 0.32 | 2795 ± 19 / 0.68 | 2880 ± 10 / 0.35 |
|  | 20 | 3771 ± 35 / 0.93 | 3865 ± 25 / 0.65 | 3930 ± 6 / 0.15 |
|  | 30 | 4521 ± 40 / 0.88 | 4507 ± 15 / 0.33 | 4682 ± 13 / 0.28 |
|  |  |  |  |  |

**Table A3.** Longitudinal moduli with standard deviations and coefficients of variation CV obtained from IET and TT for composites with PA66 and PBT matrices.

| Matrix | *w*_F_ | $E_{l}^{\mathrm{TT}}$ / CV  loading | $E_{l}^{\mathrm{TT}}$ / CV  unloading | $E_{l}^{\mathrm{IET}}$ / CV  60 mm | $E_{l}^{\mathrm{IET}}$ / CV  80 mm |
| --- | --- | --- | --- | --- | --- |
|  | % | MPa / % | MPa / % | MPa / % | MPa / % |
| PA66 | 0 | 3421 ± 11 / 0.32 | 3457 ± 53 / 1.53 | 3386 ± 11 / 0.32 | 3343 ± 15 / 0.45 |
|  | 30 | 5319 ± 99 / 1.86 | 5332 ± 102 / 1.91 | 5322 ± 23 / 0.43 | 5293 ± 18 / 0.34 |
|  | 40 | 6084 ± 101 / 1.66 | 6131 ± 100 / 1.63 | 5997 ± 16 / 0.27 | 5927 ± 23 / 0.43 |
| PBT | 0 | 2897 ± 45 / 1.55 | 2942 ± 57 / 1.94 | 2880 ± 10 / 0.35 | 2855 ± 13 / 0.46 |
|  | 20 | 3745 ± 32 / 0.85 | 3769 ± 31 / 0.82 | 3930 ± 6 / 0.15 | 3852 ± 11 / 0.29 |
|  | 30 | 4561 ± 104 / 2.28 | 4559 ± 112 / 2.46 | 4682 ± 13 / 0.28 | 4659 ± 10 / 0.21 |

**Table A4.** Torsion moduli obtained with OT (${G´}_{t}^{\mathrm{OT}})$ and IET ($G_{t}^{\mathrm{IET}})$ with STD and CV for composites with PA66 and PBT matrices.

| Matrix | *w*_F_ | ${G´}_{t}^{\mathrm{OT}}$ / CV | $G_{t}^{\mathrm{IET}}$ / CV  *l* = 60 mm | $G_{t}^{\mathrm{IET}}$ / CV  *l* = 80 mm |
| --- | --- | --- | --- | --- |
|  | % | MPa | MPa | MPa |
| PA66 | 0 | 1183 ± 25 / 2.11 | 1139 ± 12 / 1.05 | 1132 ± 9 / 0.80 |
|  | 30 | 1849 ± 33 / 1.78 | 1791 ± 1 / 0.06 | 1780 ± 15 / 0.84 |
|  | 40 | 2185 ± 92 / 4.21 | 2113 ± 16 / 0.76 | 2095 ± 20 / 0.95 |
| PBT | 0 | 960 ± 7 / 0.73 | 978 ± 5 / 0.51 | 972 ± 7 / 0.72 |
|  | 20 | 1306 ± 61 / 4.67 | 1378 ± 5 / 0.36 | 1344 ± 8 / 0.60 |
|  | 30 | 1578 ± 57 / 3.61 | 1640 ± 4 / 0.24 | 1639 ± 6 / 0.37 |
